# A Finite Element Study of Micropipette Aspiration of Single Cells: Effect of Compressibility

**DOI:** 10.1155/2012/192618

**Published:** 2012-02-09

**Authors:** Amirhossein Jafari Bidhendi, Rami K. Korhonen

**Affiliations:** Department of Applied Physics, University of Eastern Finland, P.O. Box 1627, 70211 Kuopio, Finland

## Abstract

Micropipette aspiration (MA) technique has been widely used to measure the viscoelastic properties of different cell types. Cells experience nonlinear large deformations during the aspiration procedure. Neo-Hookean viscohyperelastic (NHVH) incompressible and compressible models were used to simulate the creep behavior of cells in MA, particularly accounting for the effect of compressibility, bulk relaxation, and hardening phenomena under large strain. In order to find optimal material parameters, the models were fitted to the experimental data available for mesenchymal stem cells. Finally, through Neo-Hookean porohyperelastic (NHPH) material model for the cell, the influence of fluid flow on the aspiration length of the cell was studied. Based on the results, we suggest that the compressibility and bulk relaxation/fluid flow play a significant role in the deformation behavior of single cells and should be taken into account in the analysis of the mechanics of cells.

## 1. Introduction

Recent studies have shown that the mechanical factors play an important role in cell metabolism, differentiation, and function [1, 2]. Cells possess mechanical properties that change along with changes in cytoskeletal network in each stage of differentiation or disease [3]. Mechanobiology of tissues and cell biosynthesis are related to the mechanical signals experienced by cells, and the deformation behavior of cells within a tissue is affected by the mechanical environment and mechanical properties of cells. Hence, a method to search for the appropriate cells in a mass of cultured stem cells may be based on measuring the mechanical properties of those cells. Moreover, evaluation of the mechanical properties of cells improves the understanding of the onset and progression of tissue pathologies.

**Figure 1:**
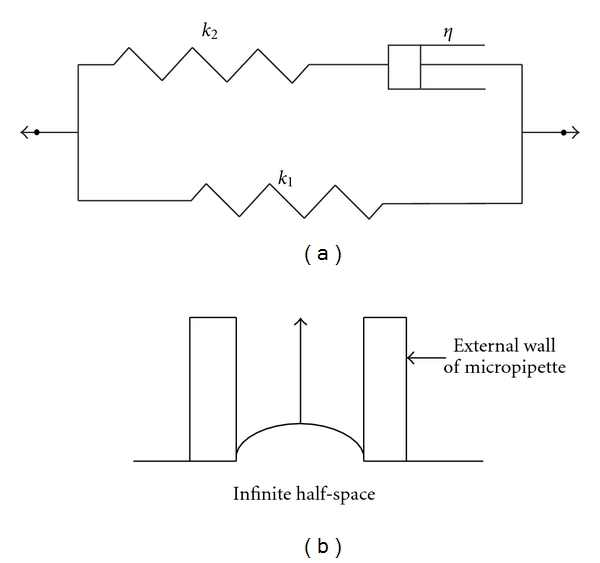
(a) Three-parameter viscoelastic linear solid for the cell and (b) infinite half-space model for the cell in MA.

Micropipette aspiration (MA) technique has been widely used to measure the mechanical properties of different cell types [1, 4–9]. In this experiment, a cell is aspirated into a micropipette by exerting a negative pressure gradient, and the aspiration length of the cell inside the micropipette is recorded as a function of time. In MA of many cell types, the cell exhibits an initial jump inside the micropipette under a constant pressure, which is then followed by a creep until reaching the equilibrium. The time-dependent deformation behavior of the cell cannot be modeled with an elastic model. An analytical solution presented by Sato et al. [4] has been commonly employed to interpret the viscoelastic parameters of the cells from experimental data considering a three-parameter solid for the cell (Figure 1(a)). In this model, the cell is assumed as an infinite, homogenous, incompressible half-space under a uniform pressure gradient (Figure 1(b)). Small strain tensors are used for the cell deformation inside the micropipette. Although this solution has been normally used to interpret the aspiration data for its simplicity, there are some limitations in this model. The semi-infinite space assumption for the cell is only accurate when the cell to micropipette radii is considerably large, and the cell is assumed as an incompressible material, the validity of which should be scrutinized. Additionally, in MA cells undergo very large strains and cells exhibit a strain hardening behavior [10, 11], which are not included in this analytical solution.

Hyperelastic models are able to account for nonlinear behavior of cells under large strains. The time-dependent deformation behavior of different cell types has been modeled using viscoelastic, poroelastic and poroviscoelastic material models [8, 12–22]. Zhou et al. [9] studied the influence of micropipette and cell geometries on the aspiration length of the cell inside the micropipette using a Neo-Hookean viscohyperelastic (NHVH) material. However, this model neglects the importance of cell compressibility, which results in different mechanical parameters estimated for the cell. Furthermore, different assumptions of compressibility and viscoelastic properties for the cells will lead to different patterns and magnitudes of stress and strain fields within the cell and cytosol when studying the coupling of external forces in cell-extracellular matrix (ECM) interaction. The compressibility may also be an indicator of the level of cell's cytoskeletal network integrity [23]. In various other studies, the cell has been either considered as a fully incompressible material or has been assumed compressible without any emphasis on its importance [8]. Furthermore, the equivalence or the similarity of the compressibility and viscoelastic bulk relaxation and the fluid flow in the nonlinear deformation behavior of the cells should be studied.

In the current study, we developed a finite element (FE) model of the cell aspiration by applying the compressible NHVH material model. Material parameters of the model were optimized by fitting the model to the experimental data of the MA of mesenchymal stem cells [5]. We investigated the effect of different material parameters and especially the cell compressibility on the creep response of the cells in MA. Finally, Neo-Hookean porohyperelastic (NHPH) material model was developed for the cell to investigate the influence of fluid flow in MA and to clarify if the compressibility and bulk relaxation in the NHVH model could be explained by intracellular fluid.

## 2. Materials and Methods

### 2.1. Material Models

The NHVH solid was used to particularly account for the nonlinear mechanical response of cells at large strains and subsequent strain-hardening phenomenon. The Neo-Hookean material model uses a general strain energy potential [24, 25],

(1) $$U\left(\overset{̅}{I_{1}},J\right)=\;C_{10}\left(\overset{̅}{I_{1}}-3\right)+\;\frac{1}{D_{1}}{\left(J-1\right)}^{2},$$

where *U* is the strain energy per unit of reference volume, *C*_10_ and *D*_1_ are temperature-dependent material parameters, and $\overset{̅}{I_{1}}\;$is the first invariant of the deviatoric left Cauchy-Green deformation tensor$\;\overset{̅}{B}$, defined as $\overset{̅}{B}=\overset{̅}{F}\cdot {\overset{̅}{F}}^{T}$. Here $\overset{̅}{F}$ is the distortion gradient, $\;\overset{̅}{F}=\;J^{1/3}\;F$, and *J* is the elastic volume ratio. The constitutive equation for the Neo-Hookean material is given by

(2) $$\sigma_{s}=\;\frac{2}{J}C_{10}\left(\overset{̅}{B}-\frac{1}{3}\mathrm{tr}\left(\overset{̅}{B}\right)I\right)+\;\frac{2}{D_{1}}\left(J-1\right)I,$$

where *σ*_*s*_ is the Cauchy stress. The material parameters are given by

(3) $$C_{10}=\frac{G_{0}}{2},\;\;D_{1}=\;\frac{3\left(1-2\nu \right)}{G_{0}\left(1+\nu \right)},$$

where *G*_0_ is the initial shear modulus and *ν* is Poisson's ratio.

Viscoelastic properties can be defined with a Prony expansion of the dimensionless shear and bulk relaxation moduli. The Prony expansion of the time-dependent shear behavior can be formulated as [24]

(4) $$g_{R}\left(t\right)=1-\overset{N}{\underset{i=1}{\sum }}{\bar{g}}_{i}^{p}\left(1-e^{-t/\tau_{i}^{G}}\right),\;\;$$

where *g*_*R*_(*t*)(*G*_*R*_(*t*)/*G*_0_) is the dimensionless shear relaxation modulus, *G*_*R*_(*t*) is the shear modulus at the time *t*, *N* is the number of terms in the Prony series, ${\bar{g}}_{i}^{p}$ is the dimensionless Prony series parameter for shear modulus, and *τ*_*i*_^*G*^ is the relaxation characteristic time. Several studies have included the shear relaxation behavior to express the time-dependent changes in cell stiffness [4–6, 9]. Similarly, the time-dependent bulk behavior of the material can be expressed in the form of

(5) $$k_{R}\left(t\right)=1-\overset{N}{\underset{i=1}{\sum }}{\bar{k}}_{i}^{p}\left(1-e^{-t/\tau_{i}^{K}}\right),$$

where *k*_*R*_(*t*) is the dimensionless bulk relaxation modulus, *τ*_*i*_^*K*^ is the relaxation characteristic time, and$\;{\bar{k}}_{i}^{p}$ is the dimensionless Prony series parameter for bulk modulus. The bulk relaxation behavior reflects the time-dependent changes in cell volumetric behavior, and it has also been recently included in modeling the cell compression [16].

Finally, the NHPH model with a Neo-Hookean hyperelastic solid matrix, defined by *C*_10*s*_ and *D*_1*s*_, fully saturated with the intracellular fluid was developed. According to the biphasic theory, the total stress in the cell is defined as [26]

(6) $$\sigma_{t}=\;-pI+\sigma_{s},$$

where *p* is the hydrostatic pressure, *σ*_*s*_  is the effective solid stress tensor which is derived from (2) and *I* is the unit tensor. Fluid flow in the cell was modeled according to Darcy's law as [24]

(7) q=−k∇P,

where *q* is the flow rate, *k* is the permeability (with the unit m^4^/Ns), and ∇*P* is the pressure gradient across the region.

### 2.2. Finite Element Analysis

**Figure 2:**
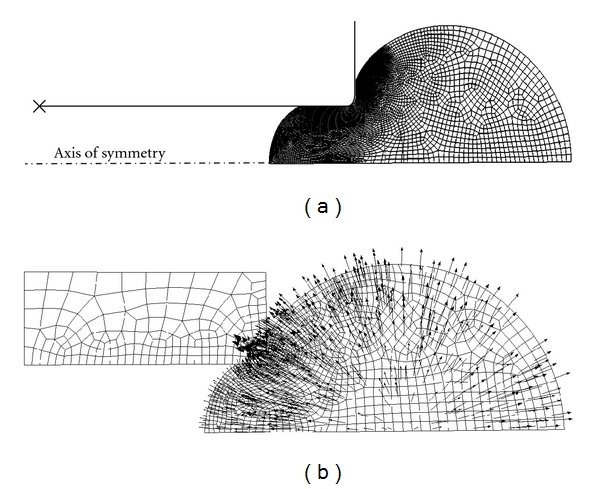
(a) The axisymmetric FE mesh of micropipette aspiration. (b) NHPH model of micropipette aspiration simulating the fluid exchange and velocity in boundary of the cell.

In the NHVH FE model of the MA, axisymmetric geometries were created for the micropipette and cell, resembling the 3-dimensional, spherical shape of the cell in suspension. The cell was considered as a homogenous continuum discretized with four-node bilinear quadratic hybrid axisymmetric elements, CAX4H (Figure 2(a)). In critical contact areas, the number of elements was increased. The micropipette was considered as analytical rigid, since it necessitates less computation time. A fillet radius was considered at the opening of the micropipette to mimic the experimentally used micropipettes [5, 9]. For a sufficiently large micropipette, such as the one used in the present study, the effect of fillet radius on the modeled response of the cell has been shown to be insignificant [9, 27]. Symmetry boundary condition was employed on the cell, disallowing the horizontal movement of the cell in the axis of symmetry. The micropipette was fixed at its reference point. Frictionless surface-to-surface contact (Abaqus/Standard, Dassault Systèmes Simulia Corp, Providence, RI, USA) was implemented between the micropipette and the cell surface. Since the inertial forces are negligible during the aspiration, the procedure was considered quasistatic. The aspiration pressure was set to 890 Pa, according to the experiments, reaching its maximum almost instantaneously (within 0.001 s). Abaqus 6.8.1 finite element package was used in all pre- and postprocessing. Sensitivity of the results to the mesh density and the element type was investigated.

For the NHPH model, in addition to the previous boundary conditions, the pore pressure at the external surface of the cell was set to zero to allow free fluid flow through the boundary. In this model, general contact formulation (Abaqus/Standard) was envisaged between the micropipette and the cell surface to allow free fluid flow in the sliding interaction boundary. Thus, the micropipette was modeled as a linearly elastic solid with a very high Young's modulus to resemble the rigid glass (in order to ensure the reliability of the results in both of the contact formulations, the FE models with elastic and rigid micropipettes were compared by means of the NHVH material for the cell. The results showed a negligible difference). Four-node axisymmetric pore fluid/stress finite elements (CAX4P) were used (Figure 2(b)), and the simulations were conducted by applying the soils consolidation analysis.

### 2.3. Optimization

To find optimum set of material parameters for the models, they were fitted to the experimental creep data [5]. For that, direct search or particle swarm optimization (PSO) methods were utilized depending on the number of optimized parameters. In the case of an incompressible NHVH model, a direct search method was employed to find the optimum material parameters. In compressible NHVH models, a PSO algorithm was used based on a code between MATLAB (R2008a, MathWorks Inc., Natick, MA, USA) and Abaqus for automatic iterative minimization of weighted sum of squared errors between experimental data points and their corresponding FE model outputs. The efficiency of this algorithm in an FE application was previously confirmed [28]. In order to reduce the optimization time, the ranges for the optimized parameters were limited (based on the estimated values for NHVH and analytical models, Table 1).

### 2.4. Parametric Study

Parametric studies were carried out to demonstrate the influence of different material parameters on the response of the cell in MA. Specifically, the effect of compressibility on the deformation behavior of the cell was simulated by varying Poisson's ratio from 0.3 to 0.5, which is a commonly reported range in the literature for different cell types [7, 8, 29, 30].

In the NHPH model, a range of permeabilities (*k* = 1 × 10^−20^–1 × 10^−11^ (m^4^/Ns) were chosen for the analysis (Table 2), covering the minimum and maximum values reported for the cell [31] and membrane [32, 33]. In the study of the contribution of fluid flow to the creep deformation of the cell, the aspiration pressure was reduced to 445 Pa to decrease the computation times. Further, the effect of Poisson's ratios (0.01, 0.2, 0.35, and 0.42, Table 2), in addition to the range of permeabilities, on creep were tested. The shear modulus was constant in these simulations (319 Pa, optimized initial shear modulus for incompressible NHVH model, Table 3).

## 3. Results

### 3.1. Optimization

**Figure 3:**
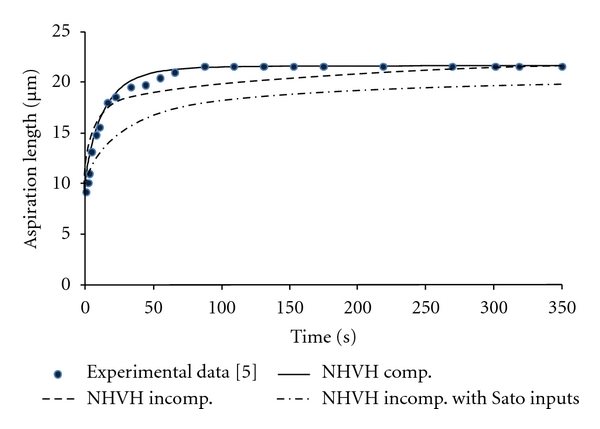
Optimized compressible and incompressible NHVH models as well as incompressible NHVH with model inputs from the literature [5] based on the Sato et al. analytical solution.

The compressible NHVH model captured well the experimental creep behavior of cells, while the incompressible NHVH model underestimated the experimental curve, especially the initial time points (Figure 3, Table 3). The incompressible NHVH model with the material parameters obtained from the literature [5] based on the Sato et al. [4] analytical solution underestimated the creep response of cells even more (Figure 3, Table 3). As compared to the incompressible NHVH model, the analytical solution overestimated the characteristic time by nearly 147%. It also underestimated the initial and infinite shear moduli by 7.5% and 1.5%, respectively (Table 3). Characteristic time, initial and infinite shear moduli differentiated by 100%, 26%, and 47% between the incompressible and compressible NHVH models, respectively (Table 3).

### 3.2. Parametric Study

**Figure 4:**
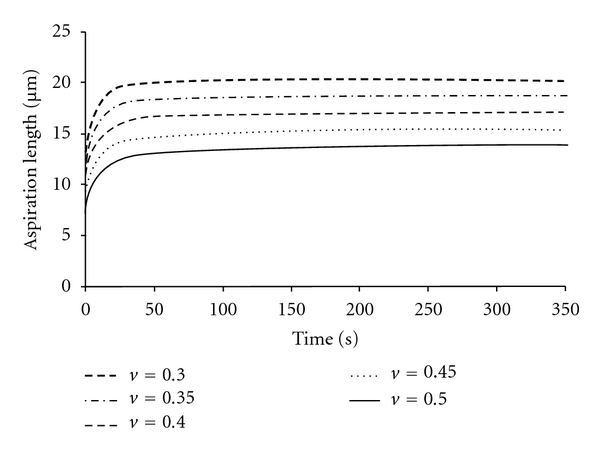
The FE results for NHVH cell model with different values of Poisson's ratio (*ν*) without bulk relaxation (*K*_*∞*_ = *K*_0_). Other parameters were Prony shear relaxation parameter ${\overset{̅}{g}}^{p}=0.51$, time constant *τ* = 3.6 (s), initial shear modulus *G*_0_ = 414 (Pa), and aspiration pressure *P* = 890 (Pa).

**Figure 5:**
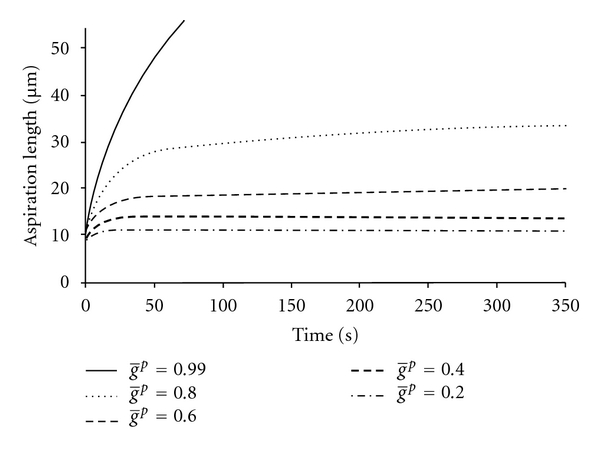
The FE results for NHVH cell model with different values of the Prony shear relaxation parameter ${\overset{̅}{g}}^{p}$. Other parameters were Prony bulk relaxation parameter ${\overset{̅}{k}}^{p}=0$, time constant *τ* = 3.6 (s), initial shear modulus *G*_0_ = 414 (Pa), Poisson's ratio *v* = 0.42, and aspiration pressure of *P* = 890 (Pa).

**Figure 6:**
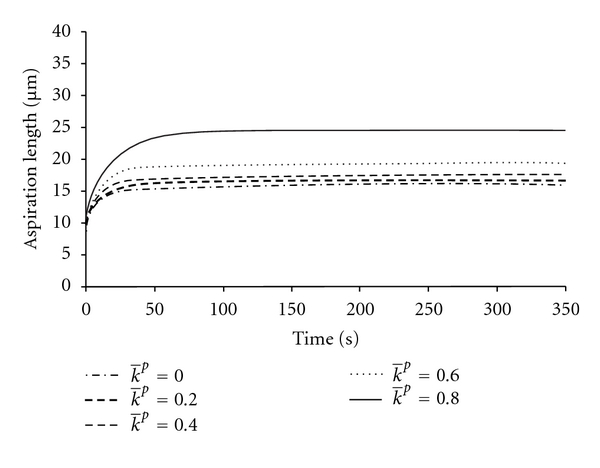
The FE results for NHVH cell model for different values of the Prony bulk relaxation parameter ${\overset{̅}{k}}^{p}$. Other parameters were ${\overset{̅}{g}}^{p}=0.4$, time constant *τ* = 3.6 (s), initial shear modulus *G*_0_ = 414 (Pa), Poisson's ratio *v* = 0.42, and aspiration pressure *P* = 890 (Pa).

**Figure 7:**
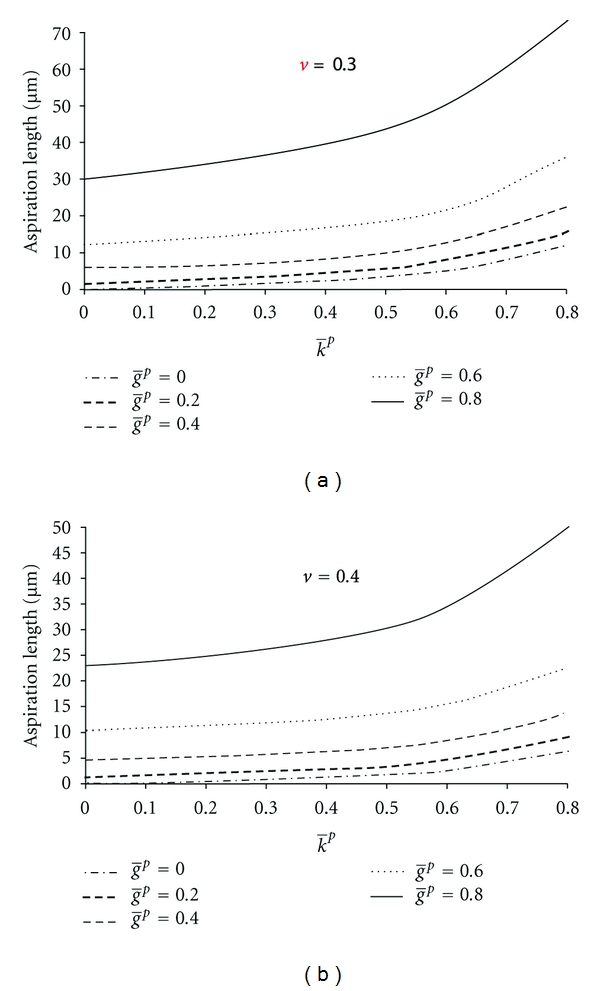
The NHVH FE model results for different values of Prony shear relaxation parameter, ${\overset{̅}{g}}^{p}$, and Prony bulk relaxation parameter, ${\overset{̅}{k}}^{p}$, with different Poisson's ratios: (a) *v* = 0.3 and (b) *v* = 0.4. Other parameters were time constant *τ* = 3.6 (s), initial shear modulus *G*_0_ = 414 (Pa), and aspiration pressure *P* = 890 (Pa). In this figure, the initial elastic jumps have been removed.

In the NHVH model, the initial and final aspiration lengths increased by 48% and 49%, respectively, by the change of Poisson's ratio from 0.5 to 0.3, while the time to reach the equilibrium remained constant  (*K*(0) = *K*(*∞*), Figure 4). By increasing ${\overset{̅}{g}}^{p}\;$(4), the cell behavior approached a Maxwell droplet (*g*_*R*_(*∞*) = 0); the aspiration length was increased and it took longer for the cell to reach the equilibrium length (Figure 5). By increasing ${\overset{̅}{k}}^{p}$ (5), the length of aspiration was increased, while the equilibrium time remained unchanged (Figure 6). With lower Poisson's ratios, the bulk relaxation had more influence on the creep deformation of the cell (zero at *v* = 0.5) (Figure 7). Furthermore, with lower Poisson's ratios, the required shear modulus to reproduce the same initial elastic jump became higher, and in contrast, a lower corresponding ${\overset{̅}{k}}^{p}$ was needed to follow the same bulk relaxation creep.

 The effect of fluid flow on the creep behavior of cells in MA was amplified by the decrease in the permeability and Poisson's ratio, as was shown by the NHPH model (Table 4). However, the creep deformation reached a plateau and was virtually the same with the permeability values lower than  1 × 10^−15^ (m^4^/Ns).

## 4. Discussion

In this study, creep behavior of a single cell in micropipette aspiration was modeled by applying incompressible and compressible NHVH material models. The model including compressibility (Poisson's ratio <  0.5) was able to capture the entire experimental aspiration curve. Although the incompressible NHVH model was able to mimic the long-term viscoelastic behavior of the cell, it failed to capture the early creep data points. Consequently, it was suggested that the creep behavior of the cell can be attributed to both shear and bulk relaxation behaviors, the latter of which is absent in incompressible assumption of the continuum cell. Thus, by taking the compressibility and bulk relaxation into account, the optimized values for viscoelastic parameters were altered. Finally, by applying the NHPH model, the fluid flow was shown to contribute to the nonlinear and time-dependent creep of the cell inside the micropipette, which is represented by bulk relaxation in the NHVH model.

The optimized material parameters for incompressible NHVH model were in good agreement with those provided by Zhou et al. [9]. However, based on the present findings, including the compressibility in the model considerably enhanced the match between the FE model and the short-term experimental creep data points. The suggested Poisson's ratio of 0.42 is interestingly close to the values estimated for chondrocytes by Trickey et al. [8], and Jones et al. [7] and for THP-1 cells studied by Lin et al. [23].

The incompressible NHVH model with the material parameters obtained by applying the Sato et al. [4] analytical solution to the experimental data [5] showed a significantly different creep response of the cell than the experimental curve. This mainly arises from strain hardening, finite cell to pipette diameter, and large deformation regimen that are not taken into account in this analytical solution.

The final length of aspiration can be attributed to both bulk and shear relaxation of the cell material (Figures 5 and 6). Clearly, the bulk relaxation modulus is more influential with lower values of Poisson's ratio, while it has no effect with Poisson's ratio of 0.5 (Figure 7). The initial shear modulus obtained from the analytical solution was lower than what was estimated for the Neo-Hookean hyperelastic model. This leads to a difference in the viscoelastic parameters between the models. In the initial phase of aspiration, the length of protrusion increases almost linearly by decreasing Poisson's ratio. By assuming the cell as an incompressible material, there is only the shear behavior to enable the deformation of the cell, and hence a lower value for this parameter is estimated as compared to the compressible cell. This also leads to a higher value estimated for dimensionless shear modulus.

For comparison between different hyperelastic models, Arruda-Boyce viscohyperelastic (ABVH) material model was also tested. In agreement with the NHVH model, the compressible ABVH model could simulate well the experimental aspiration curve. The incompressible ABVH material model with a range of values presented in the cell and tissue mechanics literature [15, 34–36] could not reach the specified length of aspiration.

In the incompressible NHVH model, there were three material parameters to be optimized; *G*_0_, ${\overset{̅}{g}}^{p}$, and *τ*. Thus, the parameters of this model could be uniquely determined by three distinct regions in the aspiration length-time curve of the cell: the initial elastic jump of the cell inside the micropipette, the equilibrium length of aspiration, and the slope of the creep curve. In the compressible NHVH model, there were two additional parameters, *D*_1_ (reflects Poisson's ratio and the initial shear modulus) and ${\overset{̅}{k}}^{p}$, which are mutually influencing each other (Figure 7), and hence they affect the uniqueness of the solution in this model. By assumption of a time-independent Poisson's ratio (${\overset{̅}{g}}^{p}={\overset{̅}{k}}^{p}$), it was possible to uniquely calculate Poisson's ratio and the initial shear modulus, as ${\overset{̅}{g}}^{p}$ was already known from the equilibrium time. However, this might not be fully realistic for viscoelastic materials in which Poisson's ratio is thought to vary with time. Even in that case, however, the optimum parameter for Poisson's ratio was always below 0.49.

The deformation-based bulk relaxation behavior may be an equivalent for the fluid flow gradient within the spongy cytoplasm [37]. In the present study, the NHPH model with a Poisson's ratio of 0.42 and permeability of less than 1.02 × 10^−15^ (m^4^/Ns) showed a 17% aspiration length change between the initial elastic and poroelastic cell elongations. This is close to the amount of contribution of bulk relaxation creep in the aspiration curve (18.5% with the optimized compressible NHVH parameters reported in Table 3 and aspiration pressure of 445 Pa). Furthermore, with a lower Poisson's ratio the effect of fluid flow was amplified. During the first seconds in MA, the NHPH model behaves as an incompressible material and the fluid flow in negligible. As a function of time during the creep, the fluid flows into lower-pressure areas. Depending on the compressibility, a large part of creep deformation was shown to be related to the volume relaxation due to fluid exchange. This result suggests that although the solid viscoelasticity, which mainly emerges from the cytoskeleton, has earlier been suggested to be the main contributor in the time-dependent cell deformation, the role of fluid may not be negligible.

Consistent with the present study, an instantaneous volume decrease was observed recently for chondrocytes in the in vivo loading of rabbit joints [38]. Furthermore, the compressibility was observed to be determinant in characterization of cell material properties and the pattern of tension distribution within the cytoplasm in compression of a cell by microplates [16]. This is in contrast with some observations in atomic force microscopy (AFM) and nanoindentation of cells where the effect of compressibility has been suggested to be insignificant [39]. Hence, the shear moduli of the cell measured with the MA technique are expected to be lower compared to their equivalents from the AFM indentation technique. This prediction is in agreement with a recent comparative study on giant phospholipid vesicles with AFM and MA techniques [40] and supported by Darling et al. [39] on Young's modulus of different mesenchymal lineage cells studied by different experimental techniques. In our study, the incompressible NHVH cell in MA technique underestimated the instantaneous and equilibrium Young's moduli by 20% and 41%, respectively, compared to the outcome of the optimized parameters for the compressible NHVH model. This is also in partial agreement with previous AFM indentation and MA of chondrocytes [6] where MA was observed to estimate 26% lower values for the equilibrium Young's modulus compared to AFM indentation technique. The apparent difference between the importance of compressibility and bulk relaxation in aspiration and elastic indentation of cells may originate from the different deformation directions the cell undergoes in each experiment and the different intracellular components involved as the load bearing components. In micropipette aspiration, the cell is mainly stretched, while in AFM test it is compressed. Based on the Tensegrity model for the cell [29] which is supported by later studies [41–43], the load bearing elements under pressure are thought to be microtubules while actin filaments bear the tensile load. The difference in mechanical properties of these subcellular elements may lead to diverging outcomes from different methods of inducing the deformation. From another point of view, the disparity may also originate from the different length and time scales of deformations in each experiment. In AFM indentation of the cells, the deformations are usually relatively small whereas in the micropipette aspiration the cell may experience large deformations/stretches. The time span of the experiment may also contribute to the discrepancy of outcomes between different techniques.

Due to the heterogeneity of the cells and anisotropic properties of the cells and membranes with compression-tension nonlinearity, the mechanical models used to interpret the results also contribute to discrepancies between the outcomes of different techniques. Evaluating the capability of material models to mimic the cell behavior in different experimental conditions can facilitate a better understanding of the mechanical behavior of cells. Eventually, the models to describe the cell behavior realistically should give the same results in all testing geometries.

The cell membrane has a very low permeability [31, 32]. In the present study, the membrane was not modeled as a separate layer, but the effect of the membrane and cell permeability on creep deformation was simulated by implementing the minimum and maximum values reported for the cell [30] and membrane [31, 32] in the homogenized cell model. Even though considering the cell membrane as a separate layer with different permeability than that of the cell would be favorable to obtain a detailed distribution of stress, strain, and fluid pressure forming within the cell and to examine pressure driven fluid flow across the membrane, the homogenized cell permeability was considered enough here.

The current model was based on a continuum assumption, and detailed intracellular elements were not included, allowing us to obtain unambiguous values of material parameters for the cell. In order to provide a detailed and realistic model of the cell that can explain the chemo-mechanical coupling of the cell-ECM interactions, the compressibility of the cytoskeleton and the contribution of the nucleus and cell membrane to the overall mechanical properties of the cell should be considered in the future. 

## 5. Conclusions

Parametric studies and optimization results suggest that compressibility and bulk relaxation behavior are two important factors in the deformation behavior of the cell in MA technique, which are not considered in the commonly used equation for MA. The compressibility of the cell that is presented by Poisson's ratio might explain the behavior of microstructural network of the cytoskeleton. Intracytoplasmic and transmembrane fluid flow could be responsible for bulk relaxation behavior and volume change of cells in MA. These mechanisms, if present in cells in vivo, may modulate significantly cell mechanics and mechanotransduction in biological tissues. Further, poroelastic fluid flow and compressibility of cells in multiscale models of biological tissues may change the prediction of cell responses in situ/in vivo.

## Figures and Tables

**Table 1:** Ranges of the material parameters allowed in optimization.

Parameter	Range
Characteristic time (*τ*)	1–7 (s)
Shear relaxation parameter $\left({\overset{̅}{g}}^{p}\right)$	0.35–0.52 (—)
Bulk relaxation parameter $\left({\overset{̅}{k}}^{p}\right)$	0.35–0.73 (—)
Initial shear modulus (*G*_0_)	319–420 (Pa)
Poisson's ratio (*v*)	0.3–0.5 (—)

**Table 2:** Ranges of the material parameters in the parametric study.

Parameter	Range
Shear relaxation parameter $\left({\overset{̅}{g}}^{p}\right)$	0.2–0.99 ( —)
Bulk relaxation parameter$\;({\overset{̅}{k}}^{p})$	0–0.8 (—)
Poisson's ratio (*v*)	0.3–0.5 (—)
Permeability (*k*)	1 × 10^−20^–1 × 10^−11^ (m^4^/Ns)
Poisson's ratio of poroelastic matrix (*v*_*s*_)	0.01, 0.2, 0.35, 0.42 (—)

**Table 3:** Optimized material parameters of the models. The values of root mean square error (RMSE) between experimental data points and corresponding points of each model are also listed.

Model	*v*	*τ* (s)	${\overset{̅}{k}}^{p}$	${\overset{̅}{g}}^{p}$	*G*_0_ (Pa)	RMSE
Incompressible NHVH with inputs from Tan et al. [5]	0.5	7.8	0	0.58	296	2.29
Incompressible NHVH	0.5	1.2	0	0.61	319	1.42
Compressible NHVH	0.42	3.6	0.7	0.51	414	0.44

Parameters for incompressible and compressible NHVH models were obtained by optimizing the FE model to the corresponding experimental curve, while the parameters for the incompressible NHVH model were also obtained from the literature [5].

**Table 4:** Influence of poroelasticity in creep of the cell into micropipette in different Poisson's ratios (*G*_0_  = 319 Pa and aspiration pressure = 445 Pa).

Poisson's ratio	Equilibrium aspiration length (*μ*m)	% Poro. at *k* = 1 × 10^−11^ (m^4^/Ns)	% Poro. at *k* = 1 × 10^−15^ (m^4^/Ns)	% Poro. at *k* = 1 × 10^−20^ (m^4^/Ns)
0.42	6.1	3.2	16.8	17.0
0.35	7.0	8.2	27.4	27.6
0.20	8.9	20.0	43.2	43.2
0.01	11.6	33.2	56.0	56.1

% Poro. = ((equilibrium  aspiration  length − initial  aspiration  length)/equilibrium  aspiration  length) × 100.
